# Genetic variants of interleukin 1B and 6 are associated with clinical outcome of surgically treated lumbar degenerative disc disease

**DOI:** 10.1186/s12891-022-05711-0

**Published:** 2022-08-13

**Authors:** Adam Biczo, Ferenc Bereczki, Kristóf Koch, Peter Pal Varga, the Genodisc Consortium, Jill Urban, Jeremy Fairbank, Chris Heywood, Sarit Sivan, Sally Roberts, Corneia Neidlinger-Wilke, Jaakko Kaprio, Michele Crites Battie, Dimitris Kletsas, Keita Ito, Jaques Huyghe, Marco Brayda-Bruno, Nevenka Kregar Velikonja, Aron Lazary

**Affiliations:** 1 Semmelweis University School of Ph.D studies, Ulloi street 26, Budapest, 1086 Hungary; 2 National Center for Spinal Disorders, Kiralyhago street 1, Budapest, 1126 Hungary; 3 Department of Spine Surgery, Department of Orthopaedics, Semmelweis University, Kiralyhago street 1, Budapest, 1126 Hungary

**Keywords:** Interleukins, Degenerative disc diseases, Long term outcome, Single nucleotide polymorphism

## Abstract

**Background:**

Successfully surgically treating degenerative disc diseases can be challenging to the spine surgeons, the long-term outcome relies on both the physical and mental status of the patient before and after treatment. Numerous studies underlined the role of inflammatory cytokines – like interleukin 1B and 6 – in the development of chronic diseases such as failed back surgery syndrome (FBSS) and major depressive disorder (MDD) which alter the outcome after spinal surgery. Our aim was to evaluate the associations of IL6 and IL1B gene polymorphisms with the long-term outcome of degenerative lumbar spine surgeries.

**Methods:**

An international genetical database (GENODISC) was combined with our institute’s clinical database to create a large pool with long term follow up data. Altogether 431 patient’s data were analysed. Patient reported outcome measures and surgical outcome was investigated in association with IL1B and IL6 SNPs with the help of ‘SNPassoc’ R genome wide association package.

**Results:**

Interleukin 1B variants analysis confirmed association with improvement of pain after surgery on individual SNP level and on haplotype level, moreover relationship with patient reported outcome and preoperative level of depression was found on individual SNP level. IL6 variants were associated with preoperative depression, somatization and with subsequent surgery.

**Conclusion:**

Understanding the complexity of spinal surgery patients’ long-term well-being is crucial in effectively treating chronic debilitating somatic diseases and the associated mental illnesses. Further studies should investigate more comprehensively the linkage of chronic physical and mental illnesses focusing on their simultaneous treatment.

**Supplementary Information:**

The online version contains supplementary material available at 10.1186/s12891-022-05711-0.

## Introduction

Degenerative disc disease (DDD) is a chronic and debilitating condition, which leads to loss of workdays in an active adult’s life [1]. Conservative treatments are mainly effective but occasionally surgical treatment is inevitable [2]. The surgical intervention in DDD aims to reduce pain and restore function. Surgical outcome is complex and multifactorial. It can be measured with objective (e.g.: muscle strength, vegetative functions) and subjective (patient reported outcome measures ‘PROMs’) assessment tools. Not uncommonly, despite the perfect surgical technique for an obvious pathology, the patient reports no significant improvement and continues to suffer from pain and even failed back surgery syndrome (FBSS) can develop [3].

Pain processing is a complex multifactorial pathway with different regulatory molecules. It has a biomedical component defined by tissue damage, an evaluative component which is influenced by coping mechanisms and an affective component which is altered by psychological disorders like depression and anxiety [4]. Cytokines and interleukins – such as interleukin 6 (IL6) and interleukin 1B (IL1B) - have an important role in regulating local pain response [5–8]. IL1B also act as an upregulator of other nociceptive agents and cascades such as IL6, prostaglandins, substance-P and matrix metalloprotease (MMP) 9 [9].

Single nucleotide polymorphisms (SNPs), the most common genetic variants have been identified in the genetic background of the development and outcome of several multifactorial diseases [10–14]. DDD is also a multifactorial entity with a strong genetic background [15] and high number of genes and their SNPs are linked to its pathomechanism [16–18]. Also genetic variations occurring in COL1A1, COL9a3 and VDR genes seems to be associated with the development of LDD [19]. DDD related pain is also influenced by different genes and their variations such as catechol-O-methyltransferase (COMT) [20] and β2-adrenergic receptor genes (ARDRB2) [5, 21]. According to published data the IL1 gene family (IL1A, IL1B, IL1RN) and the IL6 gene variations have connection with degenerative spinal pathologies [22–24], DDD related pain (low back pain, leg pain) [12, 25, 26] and the outcome of conservative treatment [27], but there is limited amount of data available about the associations of these gene variants in relation to spinal surgery outcome [28].

The current study focuses on evaluating the relationship between IL1B and IL6 gene polymorphisms and the long-term outcome of degenerative lumbar spine surgeries.

## Materials and methods

### Study population

Data were collected prospectively from adults (above the age of 18) who underwent routine, elective surgery for lumbar disc degeneration at one or two levels at a tertiary spine center. Prospective clinical data were linked with the subjects’ genetic data derived from the GENODISC multicenter international collaboration. Patients with minimum 2-year follow-up data were included into the final study cohort to explore the long-term outcome of the surgical procedures. Patients reoperated within 2 years due to a surgical site infection, proximal junctional kyphosis (PJK) or adjacent segment degeneration (ASD) as well as subjects undergoing either acute intervention because of neurological emergency or tumour surgery were excluded from the study. Surgeries were performed by board-certified orthopaedic surgeons or neurosurgeons specified in spinal surgery. Applied procedures included microdiscectomy, decompression and instrumented fusion (transforaminal lumbar interbody fusion or posterior fusion). All procedures were carried out using the standard median-sagittal posterior approach. All subjects signed a written consent form describing the scientific purpose of the systematic collection of their clinical and genetic data. The study was approved by the Scientific and Research Ethics Committee of the Medical Research Council Hungary (431/PI/2007).

### Clinical data

Patients completed standard and validated PROMs to assess their clinical status before the surgery and during the follow-up period [29, 30]. Pain was evaluated by the 10 cm long Visual Analogue Scale. Lumbar spine related function was measured with Oswestry Disability Index (ODI). Psychologic distress was measured by evaluating the level of depression and somatisation and was assessed with the Hungarian versions of Zung Depression Scale (ZDS) [30] and the Modified Somatic Perception Questionnaire (MSPQ) (Supplementary Material), respectively. Patients were asked to rate the overall outcome of the surgery using a five-category question; “helped a lot”, “helped”, ‘helped only little”, “didn’t help”, “made things worse”. To measure global treatment outcome (GTO) a dichotomous variable was generated based on these given answers. Good outcome was defined if the patient responded by ‘helped a lot, ‘helped’ and poor in case the patient replied by ‘only little’, ‘didn’t help’, ‘made things worse’ [31, 32]. Surgical outcome was considered “good” if no re-operation was performed at the index level within 2 years and “poor” if a subsequent surgery was needed within 2 years.

### Genotyping

DNA was extracted from venous blood or saliva samples using commercial. Five SNPs in IL1B and four SNPs in IL6 genes were selected for genotyping based on previous literature data [11, 33–37]. Genotyping was performed from 2007 to 2013 at the Technology Centre, Institute for Molecular Medicine Finland (FIMM), University of Helsinki using a Sequenom MassArray technology and the iPLEX Gold reagents (Sequenom Inc., San Diego, USA).

### Statistics

Allelic and genotype distributions, Hardy-Weinberg equilibrium, minor allele frequency (MAF) as well as associations between genetic variants and outcomes were determined and analysed using the 'SNPassoc' and 'haplo.stats' R software packages [38]. Genetic associations with preoperative and postoperative pain, disability, and psychological distress as well as global treatment and surgical outcome were investigated. Individual genotype-phenotype associations were studied in generalized linear models (GLM). Genetic subgroups with less than 4 (1%) subjects were excluded from subsequent statistical analyses. Haplotype-phenotype association was analysed applying haplo.score tests and GLM models. In haplo.score analysis, a global test of association as well as individual haplotype-specific tests are carried out using a score function. Disc herniation subgroup (patients underwent microdiscectomy) was also analysed separately to investigate the role of IL SNPs in sciatica. Significant covariates (age, gender, weight, height, preop ZDS and preop MSPQ score, type of surgery) were determined and entered into the models for each outcome. *P*-value less than 0.05 was considered significant.

## Results

### Study population

A total of 431 subjects (all Caucasians) met the study inclusion criteria. Mean age were 52.7 (SD:13.9y) years (from 20 to 88 years) and male/female ratio was 0.6 (male:166, female:265). As the index surgery 171 patients had microdiscectomy, 22 patients had decompression, 142 patients had one level fusion and 96 patients had 2-level fusion. In the final study cohort, 44 patients required a subsequent lumbar surgery at the index level during the follow-up. Eight patients had re-discectomy or decompression, 35 required fusion and in 1 case the implants had to be removed.

### Descriptive statistics of genotyping

**Fig. 1:**
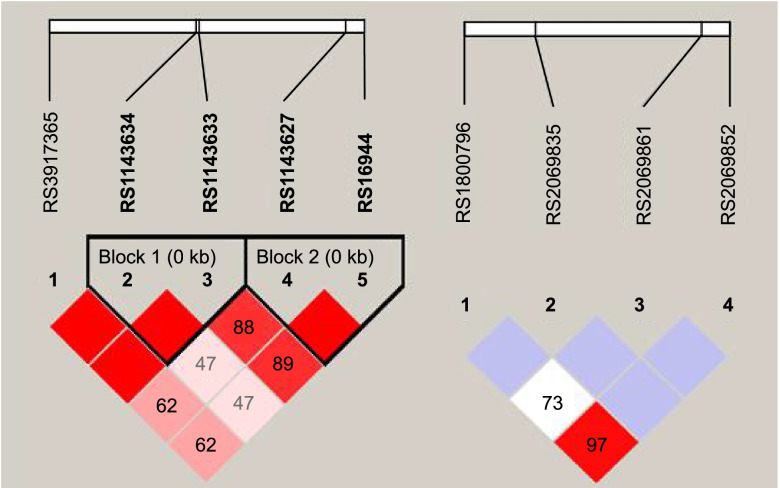
Linkage disequilibrium (LD) map of IL1B (**A**) and IL6 (**B**) SNPs. Squares are colored darker if the |D’| value is high, that is, LD is strong. Empty dark squares mean |D’|=1, that is, complete LD between two single nucleotide polymorphisms

Table 1 shows the results of the genotyping process. The genotyping success rate was more than 97% in all cases. All studied SNPs were in Hardy-Weinberg equilibrium. Two haploblock from IL1B gene were identified consisting of 2-2 SNPs *(‘rs1143634-rs1143633’*and ‘*rs1143627-rs16944’*) and no haploblock was identified on the IL6 gene as seen on Fig. 1.

**Table 1:** The descriptive statistics of the genotyped SNPs

Gene	rs number	Position	Alleles	Major allele frequency %	HWE	missing (%)
IL1B	rs3917365	3’ UTR	C/T	91.5	0.344	0.2
IL1B	rs1143634	Exon 5	C/T	73.7	1.000	0.5
IL1B	rs1143633	Intron 4	G/A	65.1	0.521	1.2
IL1B	rs1143627	Promoter	T/C	65.6	0.914	0.2
IL1B	rs16944	Promoter	G/A	65.8	1.000	2.1
IL6	rs2069852	3’ UTR	G/A	95.1	0.613	0
IL6	rs2069861	3’ UTR	C/T	93.6	0.688	0.5
IL6	rs2069835	Intron	T/C	92.7	0.264	1.4
IL6	rs1800796	Promoter	G/C	93.4	1.000	1.2

The descriptive statistics of the genotyped SNPs, HWE: Hardy-Weinberg equilibrium

### Associations of IL1B and IL6 gene variants with preoperative PROMs

In the overall population the mean±SD values of preoperative ODI score was 47.4±18.4 and the mean VAS score was 7.2±1.9, the mean ZDS was 39.6±8.1, MSPQ was 8.3±5.7. No individual SNP was associated with preoperative ODI and pain (Table 2), however both IL genes had SNPs related to the level of depression. ‘T’ allele of *rs1143627* IL1B SNP was associated with higher level of depression (ZDS was 40.6±8.7, 39.2±7.3 and 38.3±8.0 in case of ‘T/T’, ‘T/C’ and ‘C/C’ genotypes, respectively, *p-*value=0.025 in log additive model). IL1B *rs16944* ‘G’ allele carriers also showed higher level of depression (ZDS was 40.6±8.8, 39.2±7.3 and 38.0±8.0 in case of ‘G/G’, ‘A/G’ and ‘A/A’ genotypes, respectively, *p-*value=0.025 in log additive model). *rs1143634* IL1B was associated with ZDS in an overdominant model (*p*=0.025, “C/T” mean ZDS±SD was 40.8±8.4 and 39.0±7.8 in case of ‘C/T’ and ‘C/C’+’T/T’ genotype groups). The ‘C’ allele of IL6 SNP *rs2069835* was linked to increased level of depression (mean ZDS±SD were 39.2±7.8, 42.2±9.2, and 45.3±10.1 in case of ‘T/T’, ‘T/C’ and ‘C/C’ genotypes, respectively, *p=*0.003 in log-additive model).

IL6 *rs2069835* was associated with the level of preoperative somatization (Mean MSPQ±SD was 8.0±5.3 and 10.2±7.0 in case of ‘T/T’ and ‘T/C’+’C/C’ genotypes respectively, *p*=0.010, in dominant model) (Table 2).

IL1B haplotypes were not associated with preoperative ODI, depression, somatization, and pain (data not shown).

**Table 2:** Associations of IL1B and IL6 gene variants with preoperative PROMs

SNP	Genotype	Preop ZDS	***p***	Preop MSPQ	***p***	***Preop ODI***	***p***	***Preop pain***	***p***
	(N)	Mean+SD		Mean+SD		Mean+SD		Mean+SD	
IL1B_rs3917365	C/C (358) T/C (71) T/T (1)	39.5+8.2 39.6+7.1 63	*0.146*	8.3+5.8 8.2+4.9 22	*0.832*	47.5+18.7 47.1+16.7 60	*0.78*	7.2+2.0 7.2+1.9 8	*0.69*
IL1B_rs1143634	C/C (233) C/T (166) T/T (31)	39.0+7.8 40.7+8.4 38.4+7.9	0.025^**^	8.2+5.7 8.3+5.3 9.3+7.4	*0.401*	47.1+17.9 47.7+19.0 48.3+18.5	*0.655*	7.3+1.9 7.1+2.0 7.0+2.1	*0.253*
IL1B_rs1143633	G/G (184) A/G (187) A/A (41)	39.4+8.1 39.9+7.8 39.7+9.0	*0.526*	8.6+5.6 7.9+5.4 8.9+6.9	*0.183*	47.1+17.9 47.4+19.2 47.6+16.0	*0.813*	7.1+2.0 7.3+1.8 7.4+2.0	*0.170*
IL1B_rs1143627	T/T (184) T/C (196) C/C (50)	40.6+8.7 39.2+7.3 38.0+8.0	***0.025***^***ƒ***^	8.0+5.5 8.5+5.8 8.6+5.6	*0.371*	48.8+18.2 46.0+18.8 48.3+16.9	*0.135*	7.1+2.1 7.3+1.8 7.2+2.2	*0.338*
IL1B_rs16944	G/G (182) A/G (191) A/A (49)	40.6+8.8 39.2+7.3 38.0+8.0	***0.025***^***ƒ***^	8.0+5.5 8.5+5.9 8.5+5.6	*0.424*	48.6+18.2 46.3+19.0 48.5+17.0	*0.207*	7.1+2.1 7.3+1.8 7.3+2.2	*0.253*
IL6_rs2069852	G/G (389) G/A (42) A/A (0)	39.7+8.0 39.1+8.7 -	*0.629*	8.4+5.8 7.0+4.4 -	*0.192*	47.2+18.3 59.8+18.5 -	*0.375*	7.2+2.0 7.1+2.0 -	*0.623*
IL6_rs2069861	C/C (376) T/C (51) T/T (2)	39.7+8.0 39.4+8.5 33.5+0.7	*0.282*	8.5+5.8 7.3+4.7 n/a	*0.103*	47.9+18.5 43.5+16.2 26+25.5	*0.095*	7.2+1.9 7.1+2.1 5.4+4.2	*0.191*
IL6_rs2069835	C/C (4) T/C (54) T/T (367)	45.3+10.1 42.2+9.2 39.2+7.8	***0.003***^***ƒ***^	10.0+10.7 10.2+6.9 8.0+5.3	0.010^*^	39.5+31.8 48.5+18.4 47.2+18.2	*0.393*	8.1+1.3 7.2+1.6 7.2+2.0	*0.385*
IL6_rs1800796	G/G (371) G/C (54) C/C (1)	39.8+8.0 38.4+8.6 40	*0.239*	8.5+5.7 6.8+4.7 14	*0.087*	47.0+18.1 50.2+19.3 40	*0.208*	7.2+2.0 7.1+1.9 4.4	*0.149*

^*^: significant in dominant model, ^**^: significant in overdominant model, †: significant in codominant model, ^‡^: significant in recessive model, ^ƒ^: significant in log-additive model

### Associations of IL1B and IL6 gene variants with postoperative outcome

#### Change in pain and disability

**Fig. 2:**
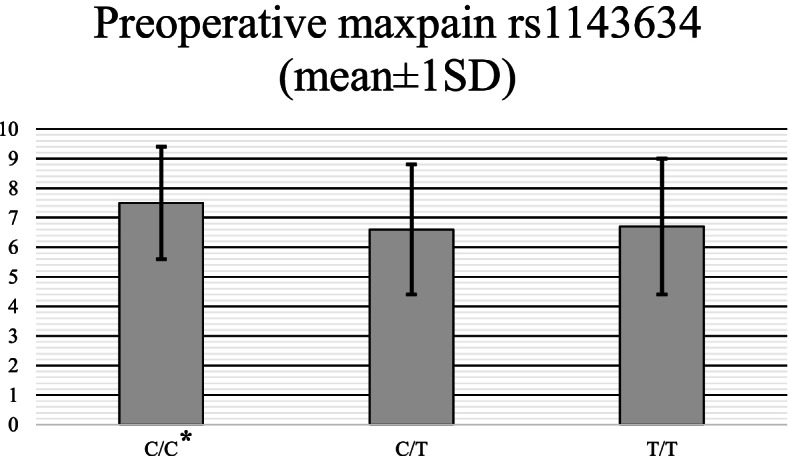
Preoperative pain in microdiscectomy subgroup (mean ±SD)

The mean overall improvement in pain intensity was 3.4±3.2 points (overall 61% improvement) in the study cohort. IL1B *rs1143633* was strongly associated with the change in the reported pain at follow-up, where the ‘A’ allele carriers had the largest improvement in pain intensity (mean change±SD (%) in pain intensity was -3.7±3.3 (50%) in ‘A/G’+’A/A’ group vs -2.9 ±3.2 (40%) in ‘G/G’ genotype, *p*=0.00085 in dominant model) (Table 3). Another IL1B SNP (*rs1143634*) was associated with sciatica in the disc herniation subgroup. In this cohort, the level of preoperative pain was significantly higher in the ‘CC’ genotype (VAS=7.5***±****1.9, 6.6±2.2 and 6.7±2.3* for ‘C/C’, ‘C/T’ and ‘T/T’ genotypes respectively, *p=*0.006 in dominant model) (Fig. 2). Change in ODI score was not associated with the studied gene variants.

**Table 3:** Associations of IL1B and IL6 gene variants with postoperative outcome

SNP	Genotype	dODI	***p***	dPAIN	***p***	GTO		***p***	Surgical outcome		***p***	***adjusted p-value***
	(N)	Mean+SD		Mean+SD		good N (%)	poor N (%)		good N (%)	poor N (%)		
IL1B_rs3917365	C/C (358) T/C (71) T/T (1)	23.1+23.6 24.5+22.23 2.2	*0.963*	-3.3+3.2 -3.7+3.3 -2.2	*0.444*	269 (76.2%) 50 (83.3%) NA	84 (13.8%) 10 (16.4%) NA	*0.861*	321 (89.6%) 64 (90.1%) 1	37 (10.4%) 7 (9.9%) 0	*0.874*	*0.951*
IL1B_rs1143634	C/C (233) C/T (166) T/T (31)	23.2+23.2 22.6+23.4 27.3+25.1	*0.353*	-3.6+3.2 -3.1+3.3 -3.4+3.3	*0.387*	175 (83.0%) 119 (80.0%) 24 (80%)	36 (17.0%) 30 (20.0%) 6 (20%)	*0.477*	212 (90.99%) 146 (87.95%) 27 (90%)	21 (9.01%) 20 (12.05%) 3 (10%)	*0.334*	*0.253*
IL1B_rs1143633	G/G (184) A/G (187) A/A (41)	22.4+.22.2 23.2+24.8 25.2+21.2	*0.311*	-2.9+3.2 -3.6+3.3 -4.1+3.2	0.00085^*^	136 (82.0%) 138 (80.7%) 44 (84.6%)	30 (18.0%) 33 (19.3%) 8 (16.4%)	*0.585*	166 (90.3%) 165 (88.2%) 52 (94.5%)	18 (9.7%) 22 (11.8%) 3 (5.5%)	*0.188*	*0.249*
IL1B_rs1143627	T/T (184) T/C (196) C/C (50)	24.4+24.0 22.0+23.6 24.3+20.4	*0.133*	-3.4+3.4 -3.3+3.0 -3.4+3.5	*0.412*	135 (78.0%) 144 (83.2%) 40 (88.9%)	38 (22.0%) 29 (16.8%) 5 (11.1%)	***0.049***^***ƒ***^	167 (90.8%) 174 (88.7%) 45 (90%)	17 (9.2%) 22 (11.3%) 5 (10%)	*0.535*	*0.556*
IL1B_rs16944	G/G (182) A/G (191) A/A (49)	24.1+24.0 22.2+23.7 24.2+20.6	*0.176*	-3.4+3.5 -3.3+3.0 -3.4+3.5	*0.486*	144 (79.1%) 155 (82.9%) 42 (89.4%)	38 (20.9%) 32 (17.1%) 5 (10.6%)	*0.056*	165 (90.66%) 169 (88.5%) 44 (89.8%)	17 (9.34%) 22 (11.5%) 5 (10.2%)	*0.505*	*0.516*
IL6_rs2069852	G/G (389) G/A (42) A/A (0)	22.9+23.0 26.3+26.5 -	*0.734*	-3.4+3.2 -3.3+3.4	*0.397*	292 (82.5%) 28 (73.7%) 0	62 (17.5%) 10 (22.3%) 0	*0.144*	346 (88.9%) 41 (97.6%)	43 (11.1%) 1 (3.4%)	***0.039***^***†***^	*0.06*
IL6_rs2069861	C/C (376) T/C (51) T/T (2)	23.7+23.7 21.1+21.3 13+12.7	*0.261*	-3.3+3.3 -3.5+2.9 -2.4+5.7	*0.360*	278 (81.3%) 39 (86.6%) 2	65 (18.7%) 6 (13.4%) 0	*0.403*	334 (88.8%) 50 (98%) 1 (50%)	42 (11.2%) 1 (2%) 1 (50%)	0.014^**^	0.004^**^
IL6_rs2069835	C/C (4) T/C (54) T/T (367)	5.1+9.7 19.9+21.6 23.9+23.5	*0.351*	-4.2+3.0 -2.9+3.1 -3.4+3.2	*0.153*	3 (75%) 36 (79.5%) 277 (83.1%)	1 (25%) 13 (20.5%) 56 (16.9%)	*0.569*	2 (50%) 46 (85.2%) 334 (88.7%)	2 (50%) 8 (14.8%) 33 (8.9%)	***0.027***^ƒ^	***0.05*** ^ƒ^
IL6_rs1800796	G/G (371) G/C (54) C/C (1)	22.7+22.8 25.7+26.4 32	*0.548*	-3.4+3.2 -3.4+3.4 -1.8	*0.486*	279 (82.5%) 36 (75.0%) 1	59 (17.5%) 12 (25.0%) 0	*0.165*	329 (88.7%) 53 (98%) 1	42 (11.3%) 1 (2%) 0	0.009^*^	0.03^*^

^*^: significant in dominant model, ^**^: significant in overdominant model, ^†^: significant in codominant model, ^‡^: significant in recessive model, ^ƒ^: significant in log-additive model

#### Global treatment outcome

In the study cohort 350 patients (82%) reported good outcome while 75 patients (17%) reported poor outcome (6 patients’ data were missing). The ‘C’ allele of IL1B *rs1143627* was related with better GTO (OR:1.49, *p=0.049* in log-additive model) (Table 3).

#### Surgical outcome

In the overall population 44 patients had poor surgical outcome (10.2%). All 4 IL6 SNPs were associated with the risk of reoperation within 2 years, even after adjusting to type of index surgery. ‘G/G’ genotype of *rs1800796* (OR:6.6, *p*=0.009, dominant model), ‘G/A’ genotype of *rs2069852* (OR:5, *p*=0.039 in codominant model) and ‘C’ allele of *rs2069835* (*p*=0.027, OR:1.27 in log-additive model) were associated with worse outcome. *rs2069861* was associated with surgical outcome in an overdominant model (*p*=0.014) (Table 3).

#### Results of haplotype analysis

There was one haploblock in IL1B gene (*rs1143634*-*rs1143633*) which was associated with change in pain. ‘C-A’ haplotype was associated with the greater improvement in pain compared to the most common ‘C-G’ haplotype (*p*= 0.001) (Table 4).

**Table 4:** IL1B haplotype association (GLM and hapscore) with the change in pain after surgery

Change in max pain GLM model					
Haploblock	Haplotype	Haplotype frequency	diff (95% CI)	hap score^**a**^	***p***
IL1B rs1143634-rs1143633	C-A T-G C-G	0.34 0.25 0.38	-0.7 (-1.2 – (-)0.3) -0.2 (-0.7 – 0.3) -3.8 (reference)	-2.46742 1.59408 0.38051	***0.001*** *0.37* *-*

^a^global *p*-value: 0.051

## Discussion

Number of spine surgeries because of DDD is continuously increasing. The rate of patients with poor outcome is between 5-70% in different surgical cohorts, and chronic pain condition because of failed back surgery syndrome (FBSS) is also not uncommon in this population. Understanding the pathophysiology of chronic pain conditions - such as FBSS - can lead clinicians to develop and apply new therapeutic methods in order to alleviate pain and improve the quality of life in this large patient group. The well-being of a patient is determined by multiple musculoskeletal, functional, and psychosocial factors [32]. Genetic influence on surgical outcome has been also highlighted by previous studies [28]. In the present study, polymorphisms of two interleukin (IL1B, IL6) genes in a large cohort of 431 patients who underwent elective lumbar spinal surgery for DDD were investigated in terms of the therapeutical outcome. Relationship between long-term treatment results, psychological factors, pain, and different IL gene variants were supported by individual SNP associations and haplotype analyses. Outcome of routine lumbar degenerative surgeries was analysed in different dimensions. Associations of IL gene variants with change in pain, disability as well as patient-reported global treatment outcome and need for a subsequent surgery were determined to elucidate the potential genetic influence.

IL1B variants were significantly related to the improvement in pain after the spine surgery, ‘A’ allele of *rs1143633* as well as ‘C-A’ haplotype of *rs1143634-rs1143633* haploblock were associated with greater improvement in pain. No other gene variant was associated with pain relieve however when we analysed the microdiscectomy subgroup we found that patients with ‘C/C’ genotype of *rs1143634* had significantly higher preoperative pain compared to the other genotypes. Other IL1B variant (*rs1143627*) was associated with patient reported global treatment outcome, while majority of the studied IL1B variants were related to the preoperative level of depression. Interestingly IL6 variants were significantly associated with the need for a subsequent surgery during the follow-up period. The ‘C’ allele of *rs2069835* IL6 SNP was associated with a higher risk for reoperation and also with increased level of preoperative depression and somatization. None of the studied gene variants were associated with preoperative spinal pain and disability level.

Number of studies supported the relationship between intervertebral disc degeneration and IL1, IL6 gene variants [23, 24, 39–42]. SNPs of these genes have been showed to be associated with the outcome of different surgical treatment [43–46], but only Moen et al. have studied the possible association of IL1 gene family and long-term outcome in patients treated because of lumbar disc herniation so far [28]. They did not find a significant relationship between *rs1143627* IL1B SNP and treatment outcome, however they did not publish the genetic effect of single SNPs but their combinations on a mixed (surgically and non-surgically treated) patient groups. The same SNP (*rs1143627)* was found to be associated with symptomatic disc herniation [26] and with DDD associated pain [25] by others. IL1B variants have been also described in association with DDD [22, 40]. IL6 variants have not been studied related to the surgical outcome of DDD yet but they were previously associated with the process of lumbar disc degeneration [24, 39, 42].

The association between IL1B, IL6 genetic variants and the therapeutic outcome after lumbar spinal surgeries can be explained by different mechanisms:

1) Progressive degeneration process can lead to persistent spinal pain and a potential indication of a subsequent surgery. IL1B is involved in multiple pathological process of disc degeneration. It stimulates extracellular matrix degradation, accelerates cellular senescence and induces apoptosis [47]. *rs1143633* in IL1B was associated with improvement of pain after surgery in our study while this SNP was found to be associated previously with the higher occurrence of disc degeneration (HIZ) [40] what can be a potential chronic pain source. IL6 variants have been also described in relation to DDD [48, 12, 42].

2) Tissue damage is often mediated through local inflammation. Inflammatory mediators such as IL6 and IL1B carry an important role in regulating and sustaining inflammation and pain. Different studies showed their potential role in disc degeneration related inflammatory process [49–51]. IL6 is crucial in homeostasis maintenance and host defence but its overproduction can cause the development or progression of diseases (such as pathologic pain) [52, 53]. The serum level of IL6 is increased in herniated disc which promotes upregulation of MMPs [53, 54]. Kraychete et al. also showed that patients with chronic low back pain due to disc herniation had higher level of serum IL6 [55]. The tissue level of IL6 can be related to the genetic variant of the gene. For example, *rs1800796* IL6 SNP (what we found to be strongly associated with FBSS) is associated with increased promoter activity boosting the local secretion of IL6 [12, 42]. The two genes have a potential influence on each other, while IL1B is described as one of the key local inducers of IL6 production [50, 51], [56]. Not surprisingly, the variants of IL1B and IL6 genes have been associated with other chronic inflammatory conditions such as periodontitis, cancer, osteoporosis, type 2 diabetes and diabetic nephropathy [33–36, 57–62].

3) Psychological issues are also important in pain response and in the development of chronic pain. Depression and anxiety have been previously described as risk factors of DDD and poor surgical outcome after spine surgeries [4, 32, 63, 64]. Interleukin genes can significantly influence the patient’s psychological profile. Chronic inflammation and dysregulation of the immune response is a key factor in the development of major depressive disorders (MDD) [65, 66]. Patients with MDD show an abnormal profile of pro- and anti-inflammatory circulating cytokines [66–69]. In animal models of MDD, increased level of pro-inflammatory cytokines caused central serotonin depletion, hypothalamic–pituitary–adrenal (HPA) axis dysregulation, microglial activation and brain structure alteration [66]. In animal inflammatory MDD (MDD-I) models, IL1B appears to be the initial triggering complex of the inflammatory cascade both centrally and peripherally [66]. In our study, some IL1B variants were significantly associated with the preoperative level of depression. These findings are in accordance with previous report about the positive association between *rs1143627* IL1B polymorphisms and MDD [70]. In accordance with our findings, Yu et al. found that the homozygotic ‘T/T’ patients of *rs1143634* had a tendency of suffering from less severe depressive symptoms than ‘T/C’ homozygotes [71]. ‘T/T’ genotype of *rs1143627* is reported to have a strong connection with major recurrent depression [72], in the meantime we found that patients with this particular genotype had worse scores on the depression scale. Two of the investigated SNPs, *rs16944* and *rs1143627* are located in the promoter region of the gene. These polymorphisms lead to altered expression of IL1B which results in local inflammation and promotes the production of MMPs [73]. A study suggested that IL1B *rs16944* gene polymorphism hinder the pharmacological response in the treatment of MDD by increasing the risk of non-remission over 6 weeks of antidepressant treatment [74]. Another IL1B SNP (*rs1143633)* was strongly associated with postoperative pain in our study while *rs1143634* was strongly associated with the preoperative pain intensity but only in the disc herniation subgroup. Previously, association of intensity of back pain and this SNP have been published in war veterans with posttraumatic stress disorder [75]. Association between *rs1143633* and pain have not been published yet, however there are a few studies investigating its relationship in paediatric MDD and schizophrenia [76, 77]. *rs2069861* in IL6 was associated with both depression and somatization in our cohort. Somatization is also an important factor in the development of symptomatic DDD [64]. Genetic variants of IL6 were linked to depression, somatization and anxiety in numerous studies [78–82].

Recently published data showed the possible role of interleukin agonist drugs in the treatment of pathological pain (e.g., chronic pain, inflammatory pain etc.) [83], therefore novel therapeutic strategies targeting IL6 or its receptors have been developed and successfully used in the treatment of selected diseases. In a paper a single intradiscal injection of tocilizumab (IL6 receptor antibody) provided short-term alleviation of discogenic pain [84]. Variants of the interleukins’ and their receptors’ genes can modify the effect of this targeted anti-inflammatory therapies, however there is no data about that so far.

In genetic association studies the sample size is highly important, as it can significantly alter the results. However, the sample size varies in the published studies, hence inconsistent genome wide association study results with non-reproducible results exist [16]. Thus, in our study we aimed to avoid sample size related study bias by using a prospective international large dataset to strengthen the findings. There are some limitations of the present study. Selection and regional population bias cannot be ruled out fully because only Caucasian patients were enrolled to the study. We did not apply any correction of the alpha-level during the genetic association testing process. We followed this method because we used a hypothesis-driven approach where effect of candidate SNPs on a phenotype was calculated. Moreover, genetic associations were tested with different statistical models (individual SNP association, haplotype analysis) to confirm the associations of the study even if the Type I error rate was not reduced. Comorbidities (e.g.: psychiatric disorders) can influence the genetic associations even if we have adjusted the statistical analyses for individual level of depression and anxiety. Therefore, study population selection bias cannot be ruled out completely. These limitations above can influence the credibility of our findings, therefore independent replications of the study are strongly recommended on different populations.

In conclusion we can state that IL1B and IL6 gene variants are associated with the psychological status and the long-term outcome of surgically treated lumbar DDD patients and these associations can be related to each other. The most plausible explanation to these findings could be linked to the major role of these cytokines in local and systemic chronic inflammation. Based on our findings and the corresponding literature advanced treatment methods could be established targeting interleukin 1B, interleukin 6 and its genes to successfully prevent/treat FBSS or even primary lumbar degenerative pathologies. On the other hand, the consideration of patient-specific genetic difference can be important to maximize the therapeutic outcome.

## Supplementary Information

**Additional file 1.**

## Data Availability

The datasets generated during the current study are not publicly available due to there is still ongoing research on the study data, but raw data are available from the corresponding author upon reasonable request.
