# Supplementary material for: Genetic variants of interleukin 1B and 6 are associated with clinical outcome of surgically treated lumbar degenerative disc disease
Source: BMC Musculoskelet Disord. 2022 Aug 13;23:774. doi: 10.1186/s12891-022-05711-0 (PMC9375337; doi:10.1186/s12891-022-05711-0)
Supplement: Supplementary file 1 — Additional file 1. [file 12891_2022_5711_MOESM1_ESM.docx]

**Methods and results of the cross-cultural adaptation and validation of the Hungarian version of Modified Somatic Perception Questionnaire**

***Methods***

**Measurement tool**

Subjects completed the Modified Somatic Perception Questionnaire (MSPQ) psychologic-condition-specific PROM, the general construct of which have been published previously several times in international literature. The MSPQ is a 13-item questionnaire where every item is a 4-point Likert scale, its overall value ranges from 0 to 39. The MSPQ scores was only calculated if all of the questions had been answered.

**Translation and cross-cultural adaptation**

The formal translation and the cross-cultural adaptation of the MSPQ into Hungarian language was based on the multistep approach suggested by Beaton et. [1]. The semantic, idiomatic, experimental, and conceptual equivalence between the source and the target questionnaire can maximally be attained through the structured adaptation process. An expert committee was formed with participating bilingual English and Hungarian language experts, spine surgeons, a physiotherapist, and a methodologist. An informed (T1-medical background, informed) and an uninformed (T2-no medical background, blinded to the concept) native Hungarian translator adapted the questionnaire into Hungarian. The translators were asked to report of any issues throughout the adaptation process. During an expert committee meeting involving both T1 and T2 the two Hungarian versions were synthetized, the reports from T1 and T2 were thoroughly discussed and a first Hungarian version was agreed on (T12). The questionnaire (T12) then was back- translated into English by two independent native English-speaking (BT1-British and BT2-US) translators who were blinded to the original English version and none of them had medical backgrounds. An expert committee meeting was held after the backtranslation process involving the translators and consensus was achieved on a prefinal Hungarian version (V1). Thereafter, a pilot study was conducted including 20 chronic LBP patients in the orthopedic inpatient clinic to test the acceptability and comprehensibility of the V1 version. The patients were interviewed about ambiguity and difficulty of the translated prefinal Hungarian V1 version. The primary goal of the pilot study was to assess how the prefinal version (V1) works in the target setting and therefore no data was collected regarding validity or reliability. A final expert committee meeting was convened and based on the recommendations and remarks of the patients a final consensus version (V2) was agreed on.

**Statistical analysis**

Internal homogeneity of the Hungarian versions of the MSPQ was tested by calculating the Cronbach-α value. Floor and ceiling effects were determined. Construct validity was demonstrated by determining the correlation between level of depression (measured by Zung Depression Scale (ZDS) score) and self-related general health (EQ-5D health VAS) as well as the psychologic subscale of EQ-5D (item 5) and the MSPQ scores. Pearson’s correlation coefficients were calculated, where r>0.40 was considered satisfactory r>0.80 as excellent (0.61–0.80 very good, 0.41–0.60 good, 0.21–0.40 fair, 0–0.20 poor). Based on the concept of the MSPQ, the pre-defined hypotheses were that the ZDS scores, EQ Health VAS and the psychologic subscale of EQ-5D expected to strongly correlate. The results of the baseline measurement (test) were used in these analyses. To examine reproducibility, we carried out test–retest analyses. Differences in the mean values of the MSPQ for the test–retest measurement was analyzed with paired t tests. Reliability was demonstrated by calculating the intraclass correlation coefficients (ICC) for absolute agreement in a two-way random effects ANOVA model. Agreement was demonstrated by determining the standard error of measurement (SEM) for the repeated measurements. The SEM was used to calculate the minimal detectable change at 95 % confidence level (MDC95%) for MSPQ applying the 2.779 SEM formula [2]. All statistical analyses were performed with SPSS 15.0 software; p values of less than 0.05 were considered significant.

***Results***

**Study cohort**

A hundred and twelve patient were recruited in the study. The first test was completed 6 months after a lumbar spinal surgery. Five participants missed the second visit and consequently did not complete the retest within fourteen days. The final study cohort consisted of 107 subjects.

**Internal consistency, floor, and ceiling effect**

Cronbach-α was 0.886 for MSPQ. No subject scored the possible maximum (39) and 7 subject (6.5%) scored the possible minimum (0) values.

**Construct validity**

The MSPQ scores correlated significantly and highly with ZDS and EQ health scores (r=0.712, p<0.000 and r=0.612, p<0.000 for ZDS and EQ health respectively). The MSPQ scores was also associated with the self-reported psychological problems measured by the EQ-5D item 5 as seen in supplementary figure 1 (p<0.000 in ANOVA model).


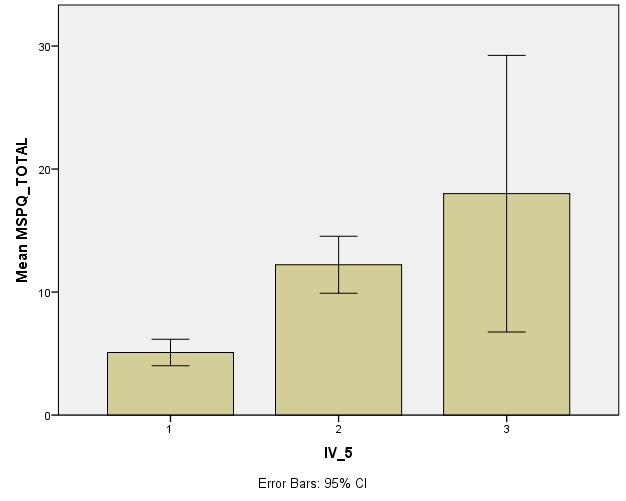
***Supplementary Figure 1***

MSPQ score association with EQ-5D item 5

**Reproducibility**

There was no significant difference between the mean MSPQ scores when the test and retest measurements were compared (mean time between the two measurements were 7.4 days). The mean±SD MSPQ score was 7.8±6.6 and 7.6±6.3 for the test-retest respectively (p=0.321). MSPQ reproducibility was confirmed by high ICC values (0.99) and relatively low SEM (0.66) value. MDC95% was approximately 1.82 points.

***Conclusion***

Based on the results of validation studies with the cross-culturally adapted Hungarian MSPQ, we can conclude that the national version of the PROM is characterized with high level of reproducibility and validity and can be used in scientific studies.

**Literature**

*1 Beaton DE, Bombardier C, Guillemin F et al (2000) Guidelines for the process of cross-cultural adaptation of self-report measures. Spine (Phila Pa 1976), 25: 3186-3191.*

*2 Terwee CB, Bot SDM, de Boer MR et al (2007) Quality criteria were proposed for measurement properties of health status questionnaires. J Clin Epidemiol 60:34–42.*
